# Royal Jelly Enhances the Ability of Myoblast C2C12 Cells to Differentiate into Multilineage Cells

**DOI:** 10.3390/molecules29071449

**Published:** 2024-03-24

**Authors:** Takumi Ito, Thira Rojasawasthien, Sachiko Yamashita Takeuchi, Hideto Okamoto, Nobuaki Okumura, Tomohiko Shirakawa, Takuma Matsubara, Tatsuo Kawamoto, Shoichiro Kokabu

**Affiliations:** 1 Division of Molecular Signaling and Biochemistry, Kyushu Dental University, Fukuoka 803-8580, Japan; r19itou@fa.kyu-dent.ac.jp (T.I.); chiab2533@gmail.com (T.R.); r21yamashita2@fa.kyu-dent.ac.jp (S.Y.T.); r15matsubara@fa.kyu-dent.ac.jp (T.M.); 2 Division of Orofacial Functions and Orthodontics, Kyushu Dental University, Fukuoka 803-8580, Japan; r16shirakawa@fa.kyu-dent.ac.jp (T.S.); r15kawamoto@fa.kyu-dent.ac.jp (T.K.); 3 Institute for Bee Products and Health Science, Yamada Bee Company, Inc., Okayama 708-0393, Japan; ho1993@yamada-bee.com (H.O.); no1780@yamada-bee.com (N.O.)

**Keywords:** C2C12, royal jelly, myogenesis, osteoblastogenesis, adipogenesis, RNA-seq, stem cell

## Abstract

Royal jelly (RJ) is recognized as beneficial to mammalian health. Multilineage differentiation potential is an important property of mesenchymal stem cells (MSCs). C2C12 cells have an innate ability to differentiate into myogenic cells. Like MSCs, C2C12 cells can also differentiate into osteoblast- and adipocyte-lineage cells. We recently reported that RJ enhances the myogenic differentiation of C2C12 cells. However, the effect of RJ on osteoblast or adipocyte differentiation is still unknown. Here in this study, we have examined the effect of RJ on the osteoblast and adipocyte differentiation of C2C12 cells. Protease-treated RJ was used to reduce the adverse effects caused by RJ supplementation. To induce osteoblast or adipocyte differentiation, cells were treated with bone morphogenetic proteins (BMP) or peroxisome proliferator-activated receptor γ (PPARγ) agonist, respectively. RNA-seq was used to analyze the effect of RJ on gene expression. We found that RJ stimulates osteoblast and adipocyte differentiation. RJ regulated 279 genes. RJ treatment upregulated glutathione-related genes. Glutathione, the most abundant antioxidative factor in cells, has been shown to promote osteoblast differentiation in MSC and MSC-like cells. Therefore, RJ may promote osteogenesis, at least in part, through the antioxidant effects of glutathione. RJ enhances the differentiation ability of C2C12 cells into multiple lineages, including myoblasts, osteoblasts, and adipocytes.

## 1. Introduction

Royal jelly (RJ) is a natural substance secreted by the cephalic glands of honeybees (e.g., *Apis mellifera*). Royal jelly is composed of water (60–70%), proteins (9–18%), sugars (7.5–23%), lipids (3–8%), and other compounds such as vitamins and minerals. The two major fatty acids in RJ are trans-10-hydroxy-2-decenoic acid (10H2DA) and 10-hydroxydecanoic acid (10HDAA), which comprise 60–80% of RJ lipids [1]. RJ provides nutrients to the queen bee throughout its lifespan and contributes to longevity and fertility. RJ is recognized as beneficial to mammalian health [2,3]. For example, RJ delays impairment of motor function during aging [4]. RJ is also used as a health supplement in humans. Previous reports showed that RJ administration led to increased hematopoietic stem-cell numbers in peripheral blood [5]. RJ administration also resulted in the alleviation of menopausal symptoms [6]. RJ contains proteins that occasionally cause anaphylactic reactions [7,8,9]. Therefore, protease-treated RJ (pRJ) was developed by treating RJ with alkaline proteases, which eliminate allergy-inducing proteins to reduce the adverse effects caused by RJ supplementation [10]. We recently reported that treatment of myoblasts with pRJ, 10H2DA, or 10HDAA, stimulated proliferation [11].

Mesenchymal stem cells (MSCs) are present in various tissues, including bone marrow, skeletal muscle, skin, fat, umbilical cord tissue, dental pulp, and periodontal tissue. MSCs are relatively easy to isolate and culture in vitro under appropriate conditions [12]. MSCs can differentiate into several mesenchymal lineage cells, such as adipocytes, osteoblasts, chondrocytes, and skeletal muscle cells. MSCs have, therefore, been used in regenerative medicine to repair bones and joints [13].

With increased aged populations worldwide, research into the aging of tissue stem cells and niches is currently attracting attention. Several factors, including cellular senescence and mitochondrial dysfunction, are involved in stem-cell aging [14]. Recently, an experiment using umbilical cord-derived MSCs revealed that RJ treatment contributes to the reduction of senescent cells and the maintenance of mitochondrial function [15]. It was also reported that RJ stimulated the secretion of extracellular vesicles in adipose-derived MSCs, leading to collagen synthesis in the dermal fibroblast [16].

Myoblast C2C12 cells were isolated from murine satellite cells, a skeletal muscle stem cell [17]. We recently reported that pRJ stimulates cell proliferation and increases the size of myotubes in vitro [18]. C2C12 cells are highly plastic and are often regarded as MSC-like cells. In addition to their potential to differentiate into myogenic cells, C2C12 cells can also differentiate into osteoblast and adipocyte lineage cells in response to an adequate stimulus [18,19].

Bone morphogenetic proteins (BMPs), members of the TGF-β superfamily, play an essential role in various biological processes [20]. Osteogenic BMPs, such as BMP-2, -4, and -7 can convert C2C12 cells from the myoblast lineage into the osteoblast lineage [18]. BMP signal transduction is initiated by the binding of the BMP ligand to two types of BMP receptors (type I and type II). Activated type-I receptors phosphorylate the C-terminal of Smad1/5 (R-Smad) in the cytoplasm. Phosphorylated R-Smads are translocated into the nucleus together with Smad4 and regulate the expression of direct target genes, such as Id-1 [21]. The peroxisome proliferator-activated receptor γ (PPARγ) belongs to the nuclear receptor superfamily and acts as a master transcription factor of adipogenesis. PPARγ agonists, such as rosiglitazone, can convert C2C12 cells into adipocyte lineage cells [19,22].

In this study, we demonstrated that pRJ treatment promoted the differentiation of C2C12 cells into both osteoblast and adipocyte lineage cells.

## 2. Results

### 2.1. pRJ Stimulates BMP-Induced Osteoblast Lineage Conversion in C2C12 Cells

**Figure 1:**
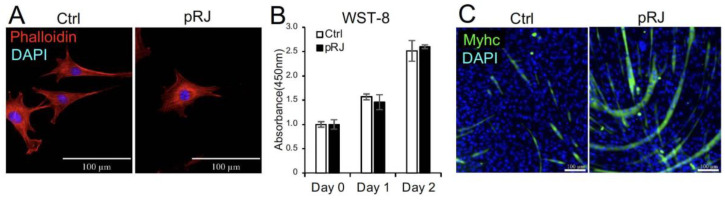
Pretreatment with pRJ stimulates myogenic differentiation in C2C12 cells. The cell nuclei and cytoskeleton were visualized by staining with DAPI (Blue) and rhodamine phalloidin (Red), respectively (**A**). The number of live C2C12 cells pretreated with or without pRJ for 1 week was assessed using the Cell Counting kit-8 on days 0, 1, and 2 (**B**). The cells were cultured in a myogenic medium for six days. Cells were then stained with an anti-myosin heavy-chain antibody (Green), and cell nuclei were visualized by staining with DAPI (Blue) (**C**). Data are presented as the mean ± SD (*n* = 3). Representative images are shown. Similar results were obtained in three independent experiments. Scale = 100 μm (**A**,**C**).

We first examined the effect of pretreatment with pRJ on the shape and viability of C2C12 cells. pRJ treatment did not significantly change cell shape or viability (Figure 1A,B). We have previously reported that pRJ enhances myogenesis in C2C12 cells [11]. In agreement with this report, C2C12 cells pretreated with pRJ showed a high potential for myogenic differentiation compared to the control group (Figure 1C).

**Figure 2:**
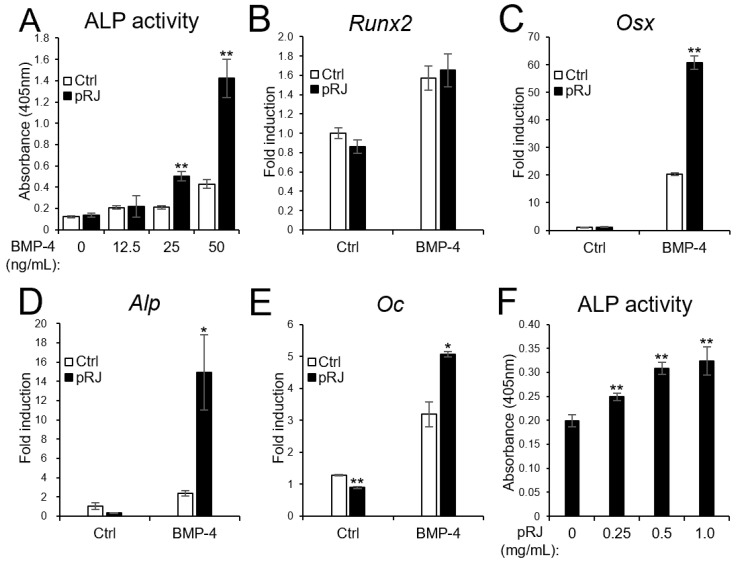
pRJ-treated cells have a high potential to differentiate into osteoblasts in response to BMPs. C2C12 cells pretreated with or without pRJ were treated with various concentrations (0, 12.5, 25, or 50 ng/mL) of rhBMP-4. ALP activity was determined on day 3 (**A**). The cells were treated with 25 ng/mL rhBMP-4. The mRNA levels of Runx2 (**B**), Osx (**C**), Alp (**D**), and Oc (**E**) were determined by qPCR on days 1 (**B**,**C**), 2 (**D**), and 3 (**E**). Non-pRJ-pretreated C2C12 cells were treated with 25 ng/mL rhBMP-4 and 0, 0.25, 0.5, or 1.0 mg/mL pRJ solution for 3 days. ALP activity was determined on day 3 (**F**). Data are presented as mean ± SD (*n* = 3). ** *p* < 0.01, * *p* < 0.05, versus control (Ctrl).

**Figure 3:**
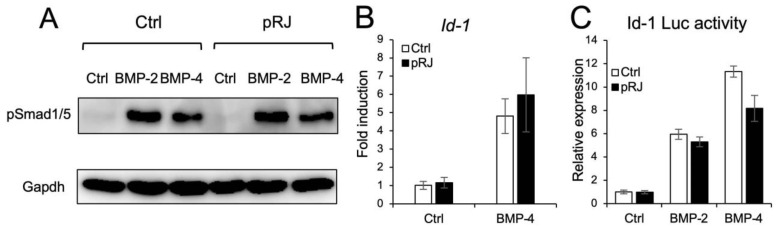
Treatment with pRJ does not affect BMP signaling. C2C12 cells pre-treated with or without pRJ were treated with 100 ng/mL rhBMP-2 or 25 ng/mL rhBMP-4 for 45 min. The protein levels of p-Smad1/5 and GAPDH were determined by Western blotting (**A**). The cells were treated with 25 ng/mL of rhBMP-4 for 1 h. The mRNA level of Id-1 was determined by qPCR (**B**). The cells were transfected with IdWT4F-luciferase reporter plasmid along with 100 ng/mL rhBMP-2 or 25 ng/mL rhBMP-4. Luciferase activity was determined on day 1 (**C**). Representative images are shown. Similar results were obtained in three independent experiments (**A**). Data are presented as mean ± SD (*n* = 3).

Next, we examined the effect of pRJ on BMP-induced osteoblast differentiation. pRJ increased alkaline phosphatase (ALP) activity, a typical marker of osteoblast differentiation, induced by BMP-4. The mRNA levels of BMP4-induced osteoblast marker genes, such as Osterix, Alp, and Osteocalcin, increased in the pRJ treatment group, although Runx2 levels did not change (Figure 2A–F). pRJ treatment increased BMP-4-induced ALP activity in a dose-dependent manner (Figure 2F), suggesting that pRJ enhances osteoblast differentiation via BMP signaling. Next, we examined whether pRJ treatment modulated BMP signal transduction. pRJ did not alter the levels of phosphorylated Smad1/5 (Figure 3A). Moreover, pRJ did not affect the mRNA level of Id-1 induced by BMP-4 (Figure 3B) nor the activity of an Id-1 luciferase reporter (Figure 3C). This suggests that pRJ does not regulate osteogenesis via a direct effect on BMP signaling.

### 2.2. pRJ Stimulates Adipocyte Lineage Conversion in C2C12 Cells

**Figure 4:**
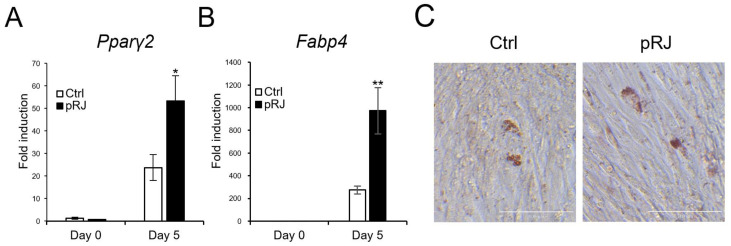
C2C12 cells pretreated with or without pRJ were treated with adipogenic medium for 5 days, and the mRNA levels of Pparγ2 (**A**) and Fabp4 (**B**) were determined by qPCR. Cells were treated with adipogenic medium for 8 days and stained with Oil Red O (**C**). Data are presented as mean ± SD (*n* = 3). ** *p* < 0.01, * *p* < 0.05, versus control (Ctrl) (**A**,**B**). Representative images are shown. Similar results were obtained in three independent experiments Scale = 100 μm (**C**).

Next, we determined the effects of pRJ on adipogenesis. When cells were treated with an adipogenic medium containing a pparγ agonist, the mRNA levels of typical adipogenic marker genes, such as Pparγ2 and Fabp4, were increased in the pRJ pretreated group (Figure 4A,B) compared to control cells. In addition, the number of cells with oil droplets was increased in the pRJ-treated group (Figure 4C), suggesting that pRJ enhanced the capacity of C2C12 cells to differentiate into adipogenic lineage cells. Overall, these data indicate that pRJ maintains the plasticity of C2C12 cells and allows them to easily differentiate into myogenic, osteogenic, and adipogenic lineages.

### 2.3. pRJ Regulated 279 Genes in C2C12 Cells

**Figure 5:**
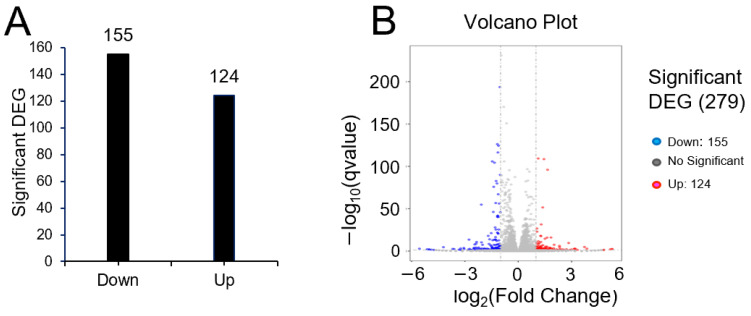
Bar graph (**A**) and differential expression volcano (**B**) of genes significantly upregulated or downregulated between the control and pRJ treatment in C2C12 cells. In the differential expression volcano plot, red dots represent genes that were significantly upregulated, and blue dots represent those that were significantly downregulated. *X*-axis—log2 fold change in gene expression. *Y*-axis—statistical significance of differential expression in log10 (q-value [fdr, padj]) (**B**).

To investigate the means by which pRJ affects plasticity, we used RNA-seq to determine global changes in gene expression in C2C12 cells treated with or without pRJ in the absence of differentiation stimuli. Treatment with pRJ significantly downregulated 155 genes and upregulated 124 genes (Supplementary Tables S1 and S2). In total, 279 genes were differentially regulated in pRJ-treated C2C12 cells (Figure 5). Genes categorized into gene ontology (GO) terms related to muscle differentiation, muscle contraction, and sarcomere assembly showed decreased expression in C2C12 cells treated with pRJ (Table 1), indicating that pRJ may prevent spontaneous differentiation. pRJ treatment also downregulated calcium-related and immune-response-related genes (Table 1). GO enrichment analysis showed that pRJ treatment upregulated the expression of glutathione-related genes (Table 2). pRJ also increased the expression of genes related to growth factor activity, such as fibroblast growth factor 1 (Fgf1), prolactin family 2 subfamily c member 3 (Prl2c3), prolactin family 2 subfamily c member 2 (Prl2c2), and betacellulin epidermal growth factor family member (Btc) (Table 2). 

**Figure 6:**
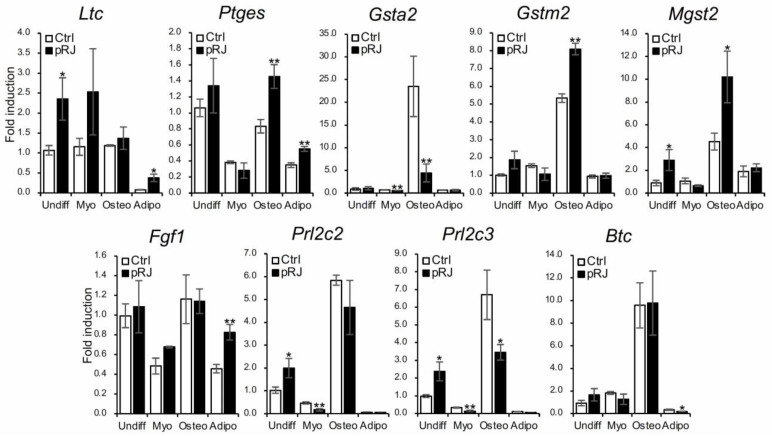
C2C12 cells pretreated with or without pRJ were treated with or without myogenic medium (Myo) for 3 days, rhBMP-4 (50 ng/mL) (Osteo) for 2 days, or adipogenic medium (Adipo) for 5 days. Ltc4s, Ptges, Gsta2, Gstm2, Mgst2, Fgf1, Prl2c3, Prl2c2, or Btc were determined by qPCR. Data are presented as mean ± SD (*n* = 3). ** *p* < 0.01, * *p* < 0.05, versus undifferentiation state (Undiff).

Finally, we used qPCR to examine the expression level of nine genes that were identified as significantly upregulated by pRJ treatment in the RNA-Seq analysis. Expression levels were examined under basal, osteogenic, myogenic, and adipogenic conditions (Figure 6). All genes were expressed to some extent at the basal level. After osteoblast differentiation, the expression of Gsta2, Gstm2, Mgst2, Prl2c2, and Prl2c3 were increased; pRJ treatment enhanced the up-regulation of Gstm2 and Mgst2 (Figure 6) following osteogenic differentiation. This suggests that the function of both Gstm2 and Mgst2 is strongly related to the effects of pRJ treatment on osteoblast differentiation. 

## 3. Discussion

In this study, pRJ enhanced the ability of C2C12 cells to differentiate into three lineages: myoblasts, osteoblasts, and adipocytes. C2C12 is derived from skeletal muscle stem cells and is capable of spontaneous muscle differentiation [17]. RNA-seq analysis showed that GO terms related to muscle differentiation, muscle contraction, and sarcomere assembly showed decreased expression in cells treated with pRJ (Table 1), suggesting that pRJ maintained cells in a proliferating and undifferentiated state in the absence of differentiation stimuli. This also suggests that cells in such a state are easy to differentiate into each lineage following adequate differentiation stimuli. 

A total of 279 genes were differentially regulated in C2C12 cells treated with pRJ. RJ regulates gene expression via global epigenetic changes [23]. pRJ increased the expression of growth factors, including Fgf1 and Btc (Table 2). Fgf1 promotes muscle satellite-cell proliferation [24]. Btc also promotes the proliferation of neural stem cells [25]. Therefore, pRJ may coordinate cell condition, at least in part, via the regulation of Fgf1 and Btc expression. RJ is a complex mixture composed of many compounds, including royalactin. Royalactin is a major protein component of RJ that induces queen-bee differentiation from honeybee larvae [26]. Royalactin also maintains pluripotency in murine embryonic stem cells [27]. However, the pRJ used in our study did not contain any protein [9]. Therefore, further experiments are needed to clarify the components that contribute to maintaining the pluripotency and plasticity of cells in the absence of royalactin. 

pRJ treatment upregulated glutathione-related genes (Table 1). Glutathione is the most abundant antioxidative factor in cells. It exerts antioxidant effects and protects cells from cytotoxic radical components and oxidative stress [28,29]. RJ has also been reported to exert antioxidant effects. A recent study suggested that 10H2DA, a major fatty acid in RJ, protected the skin against oxidative stress through the upregulation of NQO1 expression [30]. Furthermore, RJ contains phenols and other substances with antioxidant properties [31]. For instance, a previous report suggested that phenol comprises 11.1–12.0% of fresh RJ [1]. Therefore, further study is necessary to determine which specific components are responsible for antioxidant activity.

We, as well as others, have reported that polyphenols with antioxidant functions promote osteoblast differentiation in MSC and MSC-like cells [32,33,34]. Treatment of C2C12 cells with antioxidants promotes muscle differentiation [35]. Furthermore, the pretreatment of bone-marrow-derived MSCs (BMSCs) with N-acetyl-L-cysteine has been reported to increase intracellular glutathione production and cellular resistance to oxidative stress, resulting in enhanced bone regeneration [36]. In our experiment, the expression of both Gstm2 and Mgst2 was increased with osteoblast differentiation and enhanced by pRJ treatment (Figure 6). The antioxidant effects of glutathione may be involved in the promotion of osteoblastogenesis by pRJ through the regulation of Gstm2 and Mgst2 expression. In contrast, since reactive oxygen species generally promote adipocyte differentiation [37], careful further studies on antioxidant effects and adipogenesis are needed.

In addition to being a source of MSCs in bone and cartilage regenerative medicine, MSCs are thought to be involved in maintaining homeostasis by modulating the immune response. These functions have led to the use of human BMSCs in the treatment of acute graft-versus-host disease after hematopoietic stem-cell transplantation [38,39]. The use of these MSCs is expected to expand in the future, and pRJ may be useful for the quality control of MSCs. 

## 4. Materials and Methods

### 4.1. Cell Culture and Treatment

**Figure 7:**
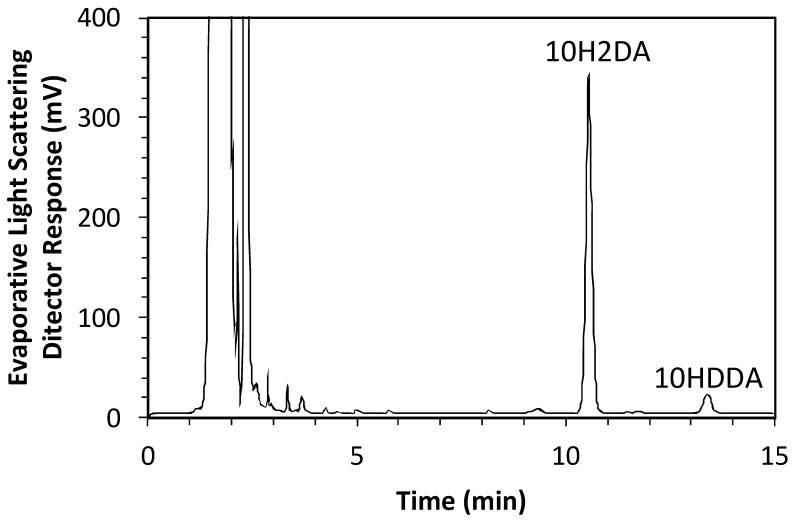
The HPLC chromatogram for the extract of pRJ powder.

Murine myoblast C2C12 cells were purchased from RIKEN (Tsukuba, Japan) in August 2022. The recognition number of the cells is RBRC-RCB0987. Cells were maintained as previously described [37]. C2C12 cells were pretreated with pRJ solution (DMEM containing 10% FBS (Nichirei Biosciences Inc., Tokyo, Japan) and 1% penicillin/streptomycin) for 7 days. Skeletal muscle differentiation in C2C12 cells was induced by culturing cells in a medium supplemented with 2% horse serum (myogenic medium) for 6 days [11]. For RNA-seq, C2C12 cells were treated with or without 1 mg/mL pRJ solution (DMEM containing 5% FBS and 1% penicillin/streptomycin) for 2 days. The pRJ (Lot.YRP-M-191126-1) containing 3.82% 10H2DA and 1.09% 10HDAA was prepared by Yamada Bee Company, Inc. (Okayama, Japan). The 10H2DA and 10HDAA in the pRJ powder were analyzed as follows. pRJ powder was extracted with methanol under agitation at room temperature. The concentration of 10H2DA and 10HDAA in the extract was measured using an HPLC system (Shimadzu, Kyoto, Japan) equipped with a Sunniest RP-AQUA column 4.6 mm ID × 150 mm (ChromaNiK Technologies Inc., Osaka, Japan) at 40 °C under a constant flow rate (1.0 mL/min) of 25% acetonitrile in 0.1% TFA (mobile phase). Detection was performed with a PDA detector at 210 nm for 10H2DA and an evaporative light scattering detector (ELSD, 35 °C) for 10HDAA. The total duration of a chromatographic run required to obtain the concentration of 10H2DA and 10HDAA was 15 min. The HPLC chromatogram for the extract can be seen in Figure 7, which was recorded by ELSD (35 °C).

### 4.2. Cell Viability Assay 

The effects of pRJ pretreatment on the viability of C2C12 cells were assessed using the WST-8 assay with the Cell Counting kit-8 assay kit (CCK-8, Dojindo, Kumamoto, Japan). Cells were cultured in 96-well plates at a density of 3.0 × 10^3^ cells/well for 1 or 2 days. Thereafter, 10 μL CCK-8 was added to each well and incubated at 37 °C for 45 min. Optical density was measured at 450 nm using a microplate reader (BioRad Laboratories, Hercules, CA, USA) [11].

### 4.3. Osteoblast Differentiation and ALP Activity Assay 

To induce osteoblast differentiation of C2C12 cells, the cells were treated with rhBMP-2 (R&D systems, Minneapolis, MN, USA) or rhBMP-4 (R&D systems) in normal DMEM supplemented with 10% FBS. C2C12 cells were cultured in the presence of 0, 0.25, 0.5, or 1.0 mg/mL pRJ solution and 25 ng/mL rhBMP-4 for 3 days. pRJ pretreated C2C12 cells were treated with rhBMP-2 or rhBMP-4 at the indicated concentrations for 3 days. After removing the culture medium, the cells were washed with phosphate-buffered saline (PBS) and treated with 0.1% Triton X-100 (Fujifilm Wako, Osaka, Japan) for 5 min. ALP activity in the cell lysate was assayed at 37 °C in a buffer containing a substrate solution composed of 0.1 M diethanolamine, 1 mM MgCl_2_, and 1 mg/mL p-nitrophenyl phosphate (Fujifilm Wako). The reaction was stopped by the addition of 3 M NaOH. The absorbance was measured at 405 nm using an iMark Microplate Absorbance Reader (BioRad Laboratories) [11].

### 4.4. Immunocytochemistry Analysis

C2C12 cells were incubated with the primary antibody for 1 h at room temperature after blocking and permeabilization with phosphate buffer saline (PBS) containing 0.3% Triton X-100 and 5% goat serum (Thermo Fisher Scientific, Waltham, MA, USA) for 30 min at room temperature. An anti-MyHC mouse monoclonal antibody (MF20, R & D systems) was used for immunocytochemical analysis. Target proteins were visualized using an Alexa 488-conjugated secondary antibody (Thermo Fisher Scientific). The cell nuclei and cytoskeletons were stained with DAPI (Dojindo) and rhodamine phalloidin (Thermo Fisher Scientific), respectively, using an ABZ-9000 (Keyence, Tokyo, Japan) microscope.

### 4.5. Western Blot Analyses 

The antibodies used for Western blot analysis were anti-phosphorated Smad1/5 rabbit (Cell signaling, Beverly, MA, USA; #9516) and HRP-conjugated anti-GAPDH mouse monoclonal antibodies, MBL, Tokyo, Japan; #M171-7). Target proteins were detected using an anti-rabbit IgG antibody conjugated with horseradish peroxidase (Cell signaling), visualized by chemiluminescence, and imaged using an LAS-4000 imaging system (Fujifilm Wako).

### 4.6. RNA Isolation and Quantitative Real-Time Polymerase Chain Reaction (qPCR) 

The FastGeneTM RNA Basic Kit (Nippon Genetics, Tokyo, Japan) was used to isolate the total RNA from the cells. A High-Capacity cDNA Reverse Transcription Kit (Applied Biosystems, Waltham, MA, USA) was used to synthesize the cDNA. SYBR green-based qPCR was performed using PowerUp SYBR (Thermo Fisher Scientific) and QuantStudio 3 Real-Time PCR System (Thermo Fisher Scientific). Relative quantification was performed using the ΔCT method with Tbp as the housekeeping gene for normalization. The following primers were used for qPCR analysis. Murine Runt-related transcription factor 2 (Runx2) (primer sequences: forward, TTCAACGATCTGAGATTTGTGGG; reverse, GGATGAGGAATGCGCCCTA), murine Osterix (Osx) (primer sequences: forward, AGAGATCTGAGCTGGGTAGAGG; reverse, AAGAGAGCCTGGCAAGAGG), murine Alkaline phosphatase (Alp) (primer sequences: forward, CGGGACTGGTACTCGGATAA; reverse, ATTCCACGTCGGTTCTGTTC), murine Osteocalcin (Oc) (primer sequences: forward, AGACTCCGGCGCTACCTT; reverse, CTCGTCACAAGCAGGGTTAAG), murine inhibitor of DNA binding 1 (Id-1) (primer sequences: forward, TTGGTCTGTCGGAGCAAAGCGT; reverse, CGTGAGTAGCAGCCGTTCATGT), murine Peroxisome Proliferator Activated Receptor γ 2 (Pparγ2) (primer sequences: forward, TGCTGTTATGGGTGAAACTCTG; reverse, CTGTGTCAACCATGGTAATTTCTT), murine Fatty acid binding protein 4 (Fabp4) (primer sequences: forward, GGATGGAAAGTCGACCACAA; reverse, TGGAAGTCACGCCTTTCATA), murine Leukotriene C4 Synthase (Ltc4s) (primer sequences: forward, CCTACAGGTGATCTCTGCACGA; reverse, TGGCGAGGAACAGCGGAAAGTA), murine prostaglandin E synthase (Ptges) (primer sequences: forward, GTGGTTTCAGCAGGGTGTCACT; reverse, GTCTTGAGTCCAGATTTGCAGCC), murine glutathione S-transferase, alpha 2 (Gsta2) (primer sequences: forward, GAGCTTGATGCCAGCCTTCTGA; reverse, TTCTCTGGCTGCCAGGATGTAG), murine glutathione S-transferase, mu 2 (Gstm2) (primer sequences: forward, AGAGCAATGCCATCCTGCGCTA; reverse, GTGTCCATAGCCTGGTTCTCCA), murine microsomal glutathione S-transferase 2 (Mgst2) (primer sequences: forward, TCGTAATGCTGTGGATGGCTGG; reverse, TTTCTCAGCGGCTTCGGCATAG), murine fibroblast growth factor 1 (Fgf1) (primer sequences: forward, CCAAGGAAACGTCCACAGTCAG; reverse, ACGGCTGAAGACATCCTGTCTC), murine prolactin family 2, subfamily c, member 3 (Prl2c3) (primer sequences: forward, TTCCTTCCAACTCCAGAAAACAAG; reverse, CTAGATCGTCCAGAGGGCTTTC), murine prolactin family 2, subfamily c, member 2 (Prl2c2) (primer sequences: forward, TTCCTTCCAACTCCAGAAAACAAG; reverse, CTAGATCGTCCAGAGGGCTTTC), murine betacellulin (Btc) (primer sequences: forward, TTCGTGGTGGACGAGCAAACTC; reverse, CCATGACCACTATCAAGCAGACC), and murine TATA-binding protein (Tbp) (primer sequences: forward, GGCGGTTTGGCTAGGTTT; reverse, GGGTTATCTTCACACACCATGA).

### 4.7. Adipogenic Medium and Oil Red O Staining

C2C12 cells were treated with an adipogenic medium (10 μM dexamethasone, 0.5 mM 3-isobutyl-1-methylxanthine, and 10 μg/mL insulin) and 10 μM rosiglitazone [40]. On day 8, adipogenic cultures of C2C12 cells were rinsed twice with PBS, fixed in 10% buffered formaldehyde, and stained with Oil Red O (Sigma Aldrich Chemicals, Burlington, MA, USA) for 10 min at room temperature.

### 4.8. Luciferase Assay

The IdWT4F-firefly luciferase reporter vector [21] and Renilla luciferase (pRL-TK) (Promega, Madison, WI, USA) were transfected into C2C12 cells using Lipofectamine 2000 (Thermo Fisher Scientific) according to the manufacturer’s instructions. Twenty-four hours after transfection, luciferase activity was measured using the Dual-Glo Luciferase Assay System (Promega). Firefly luciferase activity was normalized to the Renilla luciferase activity [11]. 

### 4.9. RNA Isolation and RNA-Seq Analysis

Total RNA was isolated from C2C12 cells using the FastGeneTM RNA Basic Kit (Nippon Genetics) according to the manufacturer’s instructions and subjected to GENEWIZ (GENEWIZ JAPAN Corp., Saitama, Japan) for RNA-seq analysis.

### 4.10. Gene-Expression Analysis

Gene expression was measured by read density; the higher the read density, the higher the level of gene expression. Gene expression was calculated using a formula that determined fragments per kilobase (kb) per million reads (FPKM) based on read counts from HT-seq (V0.6.1) [41]. The ratio of total exon fragments–mapped reads (million) was calculated as the read count mapped to the gene and normalized to the total read count. The value was then normalized to the gene length (exon length [kb]) so that the expression of genes with different sequencing depths and lengths was comparable.

### 4.11. Determination of Differentially Expressed Genes

The expression levels of all genes expressed in C2C12 cells under the two experimental treatments were compared using FPKM profiles. The input data for differential gene expression were read count data obtained from the gene-expression analysis performed using the bioconductor package edgeR (V3.4.6). The results from the edgeR analysis were further analyzed to determine the genes with significant differential expression according to the criteria of a fold change greater than 2 and a q-value (fdr, padj) less than 0.05.

### 4.12. Differential Gene GO Enrichment Analysis

GO is an international standardized gene-classification system that provides a set of dynamically updated standard vocabulary to describe the properties of genes and gene products in an organism. GO contains three ontologies that describe the molecular functions, cellular components, and biological processes of a gene. GO functional enrichment analysis returns GO terms that are enriched among differentially expressed genes against the genomic background, thus providing information on how the differentially expressed genes are related to certain biological functions. The software we used here was GOSeq [42], which is based on an extension of the hypergeometric distribution known as Wallenius’ noncentral hypergeometric distribution. This method accounts for gene length and read-count biases when performing GO analyses. The threshold for filtering was an over-represented *p*-value ≤ 0.05.

### 4.13. Statistical Analyses

Comparisons were made using unpaired analysis of variance with the Tukey–Kramer post hoc or Wilcoxon signed-rank tests. Data are presented as mean and standard deviation.

## 5. Conclusions

pRJ prevents spontaneous differentiation of C2C12 cells. In the presence of adequate stimuli, pRJ enhanced the ability of C2C12 cells to differentiate into myoblast, osteoblast, and adipocyte lineages. The antioxidant effects of glutathione may be involved in the positive effects of pRJ on osteogenesis of C2C12 cells. MSCs are expected to contribute to the future of medicine, and pRJ may be useful for the quality control of MSCs.

## Figures and Tables

**Table 1:** GO term related with down-regulated gene with pRJ treatment.

Classification	GO Term	GO ID	Gene Count	Gene Symbol
Matured muscle Muscle contraction	Actin binding	GO:0003779	11	Cnn1, Myh11, Myh3, Parvg, Avil, Tnni1, Tagln, Lmod1, Myh7, Myh1, Tnnt3
	Cytoskeleton	GO:0005856	8	Cnn1, Parvg, Cabyr, Avil, Lmod1, Actg2, Actc1, Gm2420
	Motor activity	GO:0003774	4	Myh11, Myh3, Myh7, Myh1
	Muscle–myosin complex	GO:0005859	5	Myh11, Mylpf, Myh7, Myh1, Myl9
	Structural constituent of muscle	GO:0008307	4	Myh11, Mylpf, Myh7, Myl9
	Troponin complex	GO:0005861	3	Tnnc2, Tnni1, Tnnt3
Sarcomere assembly	Z disc	GO:0030018	3	Casq1 Cacna1s Actc1
	I band	GO:0031674	15	Pvalb, Casq1, Tnnc2, Cabyr, Vldlr, Fbln7, Cabp1, Mylpf, Try5, Pcdhb12, Pcdhb4, Mmp13, Myl9
Calcium related	Calcium ion binding	GO:0005509	5	Vldlr, Cabp1, Mmp13, Tnnt3, Gm15720
	Calcium-dependent protein binding	GO:0048306	5	Cnn1, Myh11, Myh3, Myh7, Myh1
	Calcium-mediated signaling	GO:0005516	3	Edn1, Cabyr, Gm15720
Immune response	Immune response	GO:0019722	3	Prg4, Mylpf, Cxcl10
	Cellular response to interleukin-1	GO:0071347	3	Edn1, Vldlr, Ccl5

**Table 2:** GO term related with up-regulated gene with pRJ treatment.

Classification	GO Term	GO ID	Gene Count	Gene Symbol
Glutathione related	Glutathione binding	GO:00	5	Ltc4s, Ptges, Gsta2, Gstm2-ps1, Gm3776
	Glutathione transferase activity	GO:0004364	5	Ltc4s, Gsta2, Mgst2, Gstm2-ps1, Gm3776
	Glutathione metabolic process	GO:0006749	3	Gsta2, Gstm2-ps1, Gm3776
Growth factor activity	Growth factor activity	GO:0008083	4	Fgf1, Prl2c3, Prl2c2, Btc

## Data Availability

The data that support the findings of this study are available from the corresponding author upon reasonable request.

## Supplementary Materials

The following supporting information can be downloaded at: https://www.mdpi.com/article/10.3390/molecules29071449/s1.
