# Supplementary material for: Royal Jelly Enhances the Ability of Myoblast C2C12 Cells to Differentiate into Multilineage Cells
Source: Molecules. 2024 Mar 24;29(7):1449. doi: 10.3390/molecules29071449 (PMC11013243; doi:10.3390/molecules29071449)
Supplement: Supplementary file 1 [file molecules-29-01449-s001.zip › molecules-2798911-supplementary.pdf]

Supplemental Table I

	logFC	pval	GeneSymbol	Description
1	-5.563111858	5.70E-05	Gm14434	predicted gene 14434
2	-5.128647072	0.00027164	Crispld2	cysteine-rich secretory protein LCCL domain containing 2
3	-5.01736698	0.0005721	Gm2420	predicted gene 2420
4	-5.016068161	0.00054047	4930509G22Rik	RIKEN cDNA 4930509G22 gene
5	-5.016011867	0.00059412	Gm30873	predicted gene 30873
6	-5.014240087	0.00056424	Upb1	ureidopropionase beta
7	-5.013875245	0.00059014	Gm37008	predicted gene 37008
8	-4.894406731	0.00108029	Zfp583	zinc finger protein 583
9	-4.762455567	0.00217855	Pcdhb4	protocadherin beta 4
10	-4.760698926	0.0022019	Mpz	myelin protein zero
11	-4.211241378	5.13E-07	Myh11	myosin heavy polypeptide 11 smooth muscle
12	-3.688509331	9.89E-05	Ndnf	neuron-derived neurotrophic factor
13	-3.608434555	0.00017913	2510017J16Rik	RIKEN cDNA 2510017J16 gene
14	-3.337051192	0.00111269	Gja3	gap junction protein alpha 3
15	-3.245229289	2.29E-05	Try5	trypsin 5
16	-3.23220615	0.00199093	Gm26873	predicted gene 26873
17	-3.230954597	0.00206088	Gm10472	predicted gene 10472
18	-3.065574024	5.79E-06	Hoxb9	homeobox B9
19	-3.048885646	0.00015692	Gm45799	predicted gene 45799
20	-2.819544584	0.00083649	B230398E01Rik	RIKEN cDNA B230398E01 gene
21	-2.770335224	1.99E-16	Car9	carbonic anhydrase 9
22	-2.737380359	0.00010111	Gm41505	predicted gene 41505
23	-2.736837932	0.00170549	Gm11007	predicted gene 11007
24	-2.643503539	0.00281949	Gm32687	predicted gene 32687
25	-2.640656366	0.00258767	Gm20656	predicted gene 20656
26	-2.539949915	0.00055472	Cfap46	cilia and flagella associated protein 46
27	-2.491840844	4.68E-06	AC241534.1	novel transcript
28	-2.490672873	1.82E-05	Hist2h2aa2	histone cluster 2 H2aa2
29	-2.466556253	0.00094228	Cabyr	calcium-binding tyrosine-(Y)-phosphorylation regulated (fibrousheathin 2)
30	-2.449390134	1.68E-10	Cxcl10	chemokine (C-X-C motif) ligand 10
31	-2.394250473	4.56E-05	Gm11767	predicted gene 11767
32	-2.379828204	1.74E-06	A130051J06Rik	RIKEN cDNA A130051J0 gene
33	-2.371699145	2.18E-11	Mb	myoglobin
34	-2.343214788	1.58E-13	Gpr35	G protein-coupled receptor 35
35	-2.312201723	0.00304292	Parvg	parvin gamma
36	-2.28401364	1.11E-05	Wdr95	WD40 repeat domain 95
37	-2.233173335	0.00100764	Casq1	calsequestrin 1
38	-2.217949193	1.69E-06	Gm26881	predicted gene 26881
39	-2.147328352	0.00023029	Gm28071	predicted gene 28071
40	-2.145360001	0.00251446	Adra2a	adrenergic receptor alpha 2a
41	-2.098005542	0.00022936	Gm37691	predicted gene 37691
42	-2.077965203	1.50E-58	Ifit1	interferon-induced protein with tetratricopeptide repeats 1
43	-2.06931251	4.55E-13	Hist1h2al	histone cluster 1 H2al
44	-2.061947906	0.00185435	Gm28424	predicted gene 28424
45	-2.047807171	2.18E-05	Gm11189	predicted gene 11189
46	-2.010860292	5.32E-10	Ccl5	chemokine (C-C motif) ligand 5
47	-2.001386658	0.00176571	Tmem154	transmembrane protein 154
48	-1.937535736	0.00037834	Col20a1	collagen type XX alpha 1
49	-1.936137654	0.00276154	Gm13483	predicted gene 13483
50	-1.907316509	1.83E-05	Gm15720	predicted gene 15720
51	-1.89371435	0.00021482	Gm11992	predicted gene 11992
52	-1.859591647	0.0001303	Pcdhb12	protocadherin beta 12
53	-1.835621994	0.00092336	Plb1	phospholipase B1
54	-1.817403149	0.00255375	Pvalb	parvalbumin
55	-1.802729838	0.00054837	Ccdc87	coiled-coil domain containing 87
56	-1.752113258	3.45E-05	Gm5066	predicted gene 5066
57	-1.726841733	0.00239533	Lrrc24	leucine rich repeat containing 24
58	-1.712764974	0.00045024	Adgrd1	adhesion G protein-coupled receptor D1
59	-1.689772174	5.07E-21	Vldlr	very low density lipoprotein receptor
60	-1.684488259	0.00015467	Mmp13	matrix metallopeptidase 13
61	-1.646788172	4.16E-11	Itga11	integrin alpha 11
62	-1.619532149	0.00067791	Gm26910	predicted gene 26910
63	-1.612007562	7.63E-17	Isg15	ISG15 ubiquitin-like modifier
64	-1.590776699	0.00185294	Smpx	small muscle protein X-linked
65	-1.583586248	0.00164767	Creb3l4	cAMP responsive element binding protein 3-like 4
66	-1.575023322	0.00109907	P2rx3	purinergic receptor P2X ligand-gated ion channel 3
67	-1.554021322	1.69E-09	Adm	adrenomedullin
68	-1.518929934	2.37E-24	Edn1	endothelin 1
69	-1.515536259	2.30E-08	Gm9790	predicted gene 9790
70	-1.503816455	0.00032283	1810010H24Rik	RIKEN cDNA 1810010H24 gene
71	-1.477455596	2.16E-06	2610528A11Rik	RIKEN cDNA 2610528A11 gene
72	-1.467922756	4.73E-07	Gm35853	predicted gene 35853
73	-1.462764872	2.72E-110	Cnn1	calponin 1
74	-1.462048584	0.00119163	Cabp1	calcium binding protein 1
75	-1.454266444	7.74E-06	Akr1b7	aldo-keto reductase family 1 member B7
76	-1.437116539	1.16E-15	Ppfia4	protein tyrosine phosphatase receptor type f polypeptide (PTPRF) interacting protein (liprin) alpha 4
77	-1.430109503	1.06E-05	Hist1h1d	histone cluster 1 H1d
78	-1.4237724	0.001656148	1700055D18Rik	RIKEN cDNA 1700055D18 gene
79	-1.4191674	0.000274666	Sspo	SCO-spondin
80	-1.4189509	0.000506334	Lyrm7	LYR motif containing 7
81	-1.4038052	0.000146508	Lmod2	leiomodin 2 (cardiac)
82	-1.4025449	7.70E-50	Myog	myogenin
83	-1.3809019	7.54E-80	Mylpf	myosin light chain phosphorylatable fast skeletal muscle
84	-1.3749129	0.002797705	Gm4928	predicted gene 4928
85	-1.3683266	6.31E-10	Actc1	actin alpha cardiac muscle 1
86	-1.3482392	2.65E-16	Angptl6	angiopoietin-like 6
87	-1.3385061	0.000904522	Gm37589	predicted gene 37589
88	-1.3361623	8.95E-109	C1qtnf3	C1q and tumor necrosis factor related protein 3
89	-1.3347977	0.001786353	Gm16278	predicted gene 16278
90	-1.3241867	6.35E-06	Mymx	myomixer myoblast fusion factor
91	-1.299955	2.85E-31	Avil	advillin
92	-1.2955866	8.86E-05	Nkd2	naked cuticle 2
93	-1.2876125	1.50E-16	Myh3	myosin heavy polypeptide 3 skeletal muscle embryonic
94	-1.2801854	8.61E-10	Tnnc2	troponin C2 fast
95	-1.2790738	1.57E-19	Tnni1	troponin I skeletal slow 1
96	-1.2739782	0.000125884	Fzd9	frizzled class receptor 9
97	-1.269423	1.75E-06	Myh7	myosin heavy polypeptide 7 cardiac muscle beta
98	-1.2683092	1.87E-60	Slc16a3	solute carrier family 16 (monocarboxylic acid transporters) member 3
99	-1.2664855	3.48E-16	Mybph	myosin binding protein H
100	-1.2625273	1.02E-05	Lrrc51	leucine rich repeat containing 51
101	-1.2619132	0.001556228	Dyrk4	dual-specificity tyrosine-(Y)-phosphorylation regulated kinase 4
102	-1.2607892	1.08E-28	Mcam	melanoma cell adhesion molecule
103	-1.2509915	0.001667715	4930426L09Rik	RIKEN cDNA 4930426L09 gene
104	-1.249054	0.000803932	AW011738	expressed sequence AW011738
105	-1.2479599	0.001352407	Cul9	cullin 9
106	-1.2473745	0.000638094	Unc13c	unc-13 homolog C
107	-1.2447863	7.65E-87	Bnip3	BCL2/adenovirus E1B interacting protein 3
108	-1.2315062	0.002242839	5031434O11Rik	RIKEN cDNA 5031434O11 gene
109	-1.2306196	0.002283073	Gm45512	predicted gene 45512
110	-1.2189608	0.000286608	Foxf1	forkhead box F1
111	-1.2098928	8.42E-15	Map3k7cl	Map3k7 C-terminal like
112	-1.2092198	0.003104888	Cdc34b	cell division cycle 34B
113	-1.2016016	5.04E-05	Hist2h3c2	histone cluster 2, H3c2
114	-1.1998411	2.09E-05	Gm42743	predicted gene 42743
115	-1.1961254	1.13E-06	Mgarp	mitochondria localized glutamic acid rich protein
116	-1.1944505	6.77E-84	Pdk1	pyruvate dehydrogenase kinase, isoenzyme 1
117	-1.1944206	3.22E-10	Slc1a6	solute carrier family 1 (high affinity aspartate/glutamate transporter), member 6
118	-1.1809062	0.00029128	Fibin	fin bud initiation factor homolog (zebrafish)
119	-1.17557	4.46E-131	Ppp1r15a	protein phosphatase 1, regulatory subunit 15A
120	-1.1717152	0.002017704	Prg4	proteoglycan 4 (megakaryocyte stimulating factor articular superficial zone protein)
121	-1.1710786	4.47E-25	Lmod1	leiomodin 1 (smooth muscle)
122	-1.1710164	5.99E-45	Acot2	acyl-CoA thioesterase 2
123	-1.1678975	8.02E-44	Fst	follistatin
124	-1.1618348	3.44E-06	Atp6v0d2	ATPase H+ transporting lysosomal V0 subunit D2
125	-1.1572567	2.45E-05	Ypel1	yippee like 1
126	-1.1534418	1.11E-10	Myh1	myosin heavy polypeptide 1 skeletal muscle adult
127	-1.1513567	2.09E-45	Mycl	v-myc avian myelocytomatosis viral oncogene lung carcinoma derived
128	-1.1490996	2.29E-24	Tg	thyroglobulin
129	-1.143176	4.30E-121	Ddit3	DNA-damage inducible transcript 3
130	-1.1370498	0.000932658	Clec5a	C-type lectin domain family 5 member a
131	-1.1275789	5.84E-71	Actg2	actin gamma 2 smooth muscle enteric
132	-1.1275574	1.28E-23	Tnnt3	troponin T3 skeletal fast
133	-1.1203582	8.83E-44	Fbln7	fibulin 7
134	-1.1085469	4.41E-129	Myl9	myosin light polypeptide 9 regulatory
135	-1.1076884	0.000150417	Extl1	exostoses (multiple)-like 1
136	-1.1024579	9.82E-29	Mymk	myomaker myoblast fusion factor
137	-1.0946015	1.57E-15	Igfn1	immunoglobulin-like and fibronectin type III domain containing 1
138	-1.0864859	0.002082148	C330013E15Rik	RIKEN cDNA C330013E15 gene
139	-1.0841891	8.68E-08	Ankrd37	ankyrin repeat domain 37
140	-1.0744373	8.29E-60	Acta1	actin alpha 1 skeletal muscle
141	-1.0620619	1.52E-08	Ifit3	interferon-induced protein with tetratricopeptide repeats 3
142	-1.0613943	2.83E-11	Ctsf	cathepsin F
143	-1.0560986	8.81E-05	Ckm	creatine kinase muscle
144	-1.0421411	0.002420045	Osr2	odd-skipped related 2
145	-1.0399167	0.000263754	Zfr2	zinc finger RNA binding protein 2
146	-1.0328863	7.97E-199	Tagln	transgelin
147	-1.0318557	0.002301899	Fbxl22	F-box and leucine-rich repeat protein 22
148	-1.0275513	1.52E-08	Atp2a3	ATPase Ca++ transporting ubiquitous
149	-1.025185	0.001523042	Gm38020	predicted gene 38020
150	-1.020572	8.43E-08	Rasgrp3	RAS guanyl releasing protein 3
151	-1.0197325	0.000230433	Tchh	trichohyalin
152	-1.0166042	0.000166643	Cacna1s	calcium channel voltage-dependent L type alpha 1S subunit
153	-1.0134895	3.23E-94	Trib3	tribbles pseudokinase 3
154	-1.0116396	0.000975513	Arvcf	armadillo repeat gene deleted in velocardiofacial syndrome
155	-1.0050479	2.04E-49	Atf3	activating transcription factor 3

Supplemental Table I. The list of genes significantly down-regulation between control and pRJ treatment

Supplemental Table II

	logFC	pval	GeneSymbol	Description
1	5.289759639	0.000142004	AA536875	expressed sequence AA536875
2	5.289185463	0.000138061	Togaram2	TOG array regulator of axonemal microtubules 2
3	5.186942433	0.000287881	Gm45879	predicted gene 45879
4	4.82010017	0.002177338	EU599041	expressed sequence EU599041
5	4.819939088	0.002195846	Gm9918	predicted gene 9918
6	4.819524405	0.002135773	Gm13477	predicted gene 13477
7	4.81773424	0.00215836	Mir3058	microRNA 3058
8	4.812365945	0.003112793	Gm29791	predicted gene 29791
9	3.866628701	4.62E-05	Gm12606	predicted gene 12606
10	3.730358784	3.13E-07	Slc14a1	solute carrier family 14 (urea transporter) member 1
11	3.332103744	0.002015254	Gm36948	predicted gene 36948
12	3.223689827	7.65E-05	Gm13071	predicted gene 13071
13	3.169282813	4.15E-05	Zfp966	zinc finger protein 966
14	3.154224407	0.000137619	Slc25a48	solute carrier family 25 member 48
15	3.005085382	0.00047489	5930409G06Rik	RIKEN cDNA 5930409G06 gene
16	2.909176647	0.002007749	Gm20743	predicted gene 20743
17	2.840625342	2.89E-12	Sh2d1b1	SH2 domain containing 1B1
18	2.747926558	0.002560988	Gm49293	predicted gene 49293
19	2.717131834	0.000322502	Card14	caspase recruitment domain family member 14
20	2.575462579	0.000956883	AC166779.2	ribosomal protein L5 (Rpl5) pseudogene
21	2.473558219	0.000373834	C9	complement component 9
22	2.419162645	0.002816837	Esr2	estrogen receptor 2 (beta)
23	2.351116475	0.000231921	Gm13717	predicted gene 13717
24	2.342331378	0.001381173	Mir29a	microRNA 29a
25	2.331763959	5.98E-07	Unc93a2	unc-93 homolog A2
26	2.319923837	1.31E-08	Lrrc15	leucine rich repeat containing 15
27	2.276219837	0.001785632	Ccdc180	coiled-coil domain containing 180
28	2.203194877	0.002849629	Fgf1	fibroblast growth factor 1
29	2.202929338	0.002832825	Rny1	RNA Y1 small cytoplasmic Ro-associated
30	2.15577925	0.000462915	Ltc4s	leukotriene C4 synthase
31	2.049638733	0.000983241	Mannr	Mecom adjacent non-protein coding RNA
32	2.047576536	0.002721355	Clec1a	C-type lectin domain family 1 member a
33	2.022687348	4.63E-10	Greb1	gene regulated by estrogen in breast cancer protein
34	1.98870015	1.13E-05	Acot1	acyl-CoA thioesterase 1
35	1.948996717	0.000922722	A730091E23Rik	RIKEN cDNA A730091E23 gene
36	1.868743685	3.31E-05	Cftr	cystic fibrosis transmembrane conductance regulator
37	1.867164802	0.00011859	5830411N06Rik	RIKEN cDNA 5830411N06 gene
38	1.843336811	5.26E-19	Mgst2	microsomal glutathione S-transferase 2
39	1.798737301	9.09E-06	Fgd2	FYVE%2C RhoGEF and PH domain containing 2
40	1.789889633	2.41E-06	Slc6a2	solute carrier family 6 (neurotransmitter transporter noradrenalin) member 2
41	1.77884671	1.02E-05	Kbtbd12	kelch repeat and BTB (POZ) domain containing 12
42	1.765214624	0.002097006	Ldhal6b	lactate dehydrogenase A-like 6B
43	1.704338834	0.001881397	Nxpe4	neurexophilin and PC-esterase domain family member 4
44	1.700436587	6.45E-06	Hmgcll1	3-hydroxymethyl-3-methylglutaryl-Coenzyme A lyase-like 1
45	1.698197675	0.00109517	B230312C02Rik	RIKEN cDNA B230312C02 gene
46	1.654862298	0.002901034	Large2	LARGE xylosyl- and glucuronyltransferase 2
47	1.654231487	2.36E-100	Prl2c2	prolactin family 2 subfamily c member 2
48	1.626218894	6.56E-19	Fetub	fetuin beta
49	1.613559497	9.95E-08	Gm6565	predicted gene 6565
50	1.578786958	0.000504806	Hs3st5	heparan sulfate (glucosamine) 3-O-sulfotransferase 5
51	1.576567773	8.93E-06	S100a14	S100 calcium binding protein A14
52	1.55221692	4.96E-05	Gm45221	predicted gene 45221
53	1.54072313	9.73E-08	Plet1	placenta expressed transcript 1
54	1.505796195	1.39E-17	Gm42793	predicted gene 42793
55	1.499690566	3.61E-05	F2rl1	coagulation factor II (thrombin) receptor-like 1
56	1.488530238	0.001861351	Gm4813	predicted gene 4813
57	1.485523307	3.12E-06	Fsbp	fibrinogen silencer binding protein
58	1.460742207	0.001656242	Klra2	killer cell lectin-like receptor subfamily A member 2
59	1.448980823	4.62E-113	Aldh3a1	aldehyde dehydrogenase family 3 subfamily A1
60	1.447114715	6.67E-05	Arap3	ArfGAP with RhoGAP domain ankyrin repeat and PH domain 3
61	1.443016261	0.000217772	B230208H11Rik	RIKEN cDNA B230208H11 gene
62	1.430834847	0.000112139	Apob	apolipoprotein B
63	1.419598662	0.001253709	Itgal	integrin alpha L
64	1.414729024	0.001181552	Gm2788	predicted gene 2788
65	1.406115772	9.57E-05	Gm10451	predicted gene 10451
66	1.392715857	0.00014667	Anks1b	ankyrin repeat and sterile alpha motif domain containing 1B
67	1.384754032	1.70E-09	Slpi	secretory leukocyte peptidase inhibitor
68	1.378629432	3.38E-55	Prl2c3	prolactin family 2 subfamily c member 3
69	1.368434148	5.47E-05	Atp6v1c1	ATPase H+ transporting lysosomal V1 subunit C1
70	1.360901358	0.000754156	Ptges	prostaglandin E synthase
71	1.359455257	1.29E-05	Dpt	dermatopontin
72	1.356604007	0.002948617	2810039B14Rik	RIKEN cDNA 2810039B14 gene
73	1.355712923	0.003105986	Gm44949	predicted gene 44949
74	1.342646139	9.91E-05	Gm26621	predicted gene 26621
75	1.329527569	0.000603073	Gzmd	granzyme D
76	1.31630344	9.59E-09	Hba-a1	hemoglobin alpha adult chain 1
77	1.305387014	0.000442006	Gm11423	predicted gene 11423
78	1.293599081	6.96E-35	Gsta2	glutathione S-transferase alpha 2 (Yc2)
79	1.292460332	0.000175232	Rnf183	ring finger protein 183
80	1.28614275	0.003014813	Traf1	TNF receptor-associated factor 1
81	1.283950549	1.21E-05	Snora43	small nucleolar RNA H/ACA box 43
82	1.27860575	8.15E-21	Ifitm1	interferon induced transmembrane protein 1
83	1.275856997	1.35E-13	Junos	jun proto-oncogene opposite strand
84	1.2753669	2.12E-07	Ptgfr	prostaglandin F receptor
85	1.274223514	0.00110485	Gfra2	glial cell line derived neurotrophic factor family receptor alpha 2
86	1.262042932	4.51E-21	Csf2rb	colony stimulating factor 2 receptor beta low-affinity (granulocyte-macrophage)
87	1.249046563	0.002548288	Gm30624	predicted gene 30624
88	1.246837212	3.14E-05	Slc15a3	solute carrier family 15 member 3
89	1.244566829	0.000895725	Gm5176	predicted gene 5176
90	1.241478827	1.55E-07	Cd300lb	CD300 molecule like family member B
91	1.219222185	0.000292553	Gm23201	predicted gene 23201
92	1.218046678	0.000182244	Gm3776	predicted gene 3776
93	1.211603238	1.49E-06	Vezf1	vascular endothelial zinc finger 1
94	1.201983867	0.003137881	Lrrc10b	leucine rich repeat containing 10B
95	1.195018394	7.69E-06	Fgfbp1	fibroblast growth factor binding protein 1
96	1.188545558	0.000667998	Kcna3	potassium voltage-gated channel shaker-related subfamily member 3
97	1.188218459	0.001275309	5930420M18Rik	RIKEN cDNA 5930420M18 gene
98	1.187522895	2.99E-08	Loxhd1	lipoxygenase homology domains 1
99	1.182699297	4.23E-12	Gm19589	predicted gene 19589
100	1.175394943	3.83E-06	Gm13577	predicted gene 13577
101	1.17208512	0.000446019	Lctl	lactase-like
102	1.156936104	3.89E-05	Gm47507	predicted gene 47507
103	1.154656043	0.0022034	Tmem134	transmembrane protein 134
104	1.144575094	4.99E-11	Cck	cholecystokinin
105	1.133348851	2.27E-14	Btc	betacellulin epidermal growth factor family member
106	1.127183547	6.90E-114	Nqo1	NAD(P)H dehydrogenase quinone 1
107	1.125017141	9.05E-05	Sema4a	sema domain immunoglobulin domain (Ig) transmembrane domain (TM) and short cytoplasmic domain (semaphorin) 4A
108	1.11262774	1.65E-05	Tas1r2	taste receptor type 1 member 2
109	1.107408685	0.002229086	Kcnj16	potassium inwardly-rectifying channel subfamily J member 16
110	1.106503622	0.002176508	Gm9958	predicted gene 9958
111	1.104870356	0.002938765	Gm37249	predicted gene 37249
112	1.07280691	0.000410018	Trdc	T cell receptor delta constant region
113	1.070177588	3.18E-09	Cldn15	claudin 15
114	1.064168114	3.25E-05	Gm17690	predicted gene 17690
115	1.060042436	3.80E-26	Fam167a	family with sequence similarity 167 member A
116	1.0546878	2.80E-07	Serpinb9e	serine (or cysteine) peptidase inhibitor clade B member 9e
117	1.053230077	5.76E-30	Spon2	spondin 2 extracellular matrix protein
118	1.050498957	0.000722986	Gm20257	predicted gene 20257
119	1.049749548	0.002173216	Gm9797	predicted pseudogene 9797
120	1.03613052	2.18E-07	Enpp2	ectonucleotide pyrophosphatase/phosphodiesterase 2
121	1.028308364	3.74E-12	Phex	phosphate regulating endopeptidase homolog X-linked
122	1.018499914	3.72E-07	Gstm2-ps1	glutathione S-transferase mu 2 (muscle) pseudogene 1
123	1.013672775	0.000452935	Calcb	calcitonin-related polypeptide beta
124	1.0067981	0.001701733	Xk	X-linked Kx blood group

Supplemental Table II. The list of genes significantly up-regulation between control and pRJ treatment
